# Supplementary material for: Oncologists’ views on ototoxicity monitoring in head and neck cancer patients: A South Indian qualitative study
Source: PLoS One. 2025 Jan 3;20(1):e0312847. doi: 10.1371/journal.pone.0312847 (PMC11698418; doi:10.1371/journal.pone.0312847)
Supplement: Attachment — Submitted filename: PONE-D-24-14073 (1)LG.pdf [file pone.0312847.s003.pdf]

PLOS ONE

Current practices and perspectives of oncologists regarding ototoxicity monitoring in individuals with head and neck cancers undergoing radiation therapy and chemoradiotherapy: A qualitative study in a South Indian district

--Manuscript Draft--

**Manuscript Number:**	PONE-D-24-14073
**Article Type:**	Research Article
**Full Title:**	Current practices and perspectives of oncologists regarding ototoxicity monitoring in individuals with head and neck cancers undergoing radiation therapy and chemoradiotherapy: A qualitative study in a South Indian district
**Short Title:**	Oncologist’s perspectives of ototoxicity monitoring
**Corresponding Author:**	Jayashree Seethapathy<br>Sri Ramachandra Institute of Higher Education and Research (Deemed to be University)<br>Chennai, Tamil Nadu INDIA
**Keywords:**	Perspectives; ototoxicity monitoring; oncologists; head and neck cancer; qualitative; semi-structured interviews; South India
**Abstract:**	Aim<br><br>The current study aims to explore the oncologist’s awareness and perspectives of ototoxicity and ototoxicity monitoring for individuals with head and neck cancer in a South Indian district, using qualitative semi-structured interviews.<br><br>Method<br><br>The study utilized the COREQ Checklist to guide the method of the current study. A conceptual framework was developed for the formulation of the interview guides. Three medical oncologists and six radiation oncologists from tertiary centres pioneering in cancer care participated in the study. The interviews were audio recorded and transcribed verbatim. Thematic analysis was carried out using a hybrid inductive-deductive approach to present the findings under the respective over-arching themes.<br><br>Results<br><br>All oncologists are aware of the ototoxic effects caused by radiation therapy and chemoradiotherapy. It was consistently reported that the severity of the hearing loss was not substantial enough to warrant significant concern. Ototoxicity is not emphasized during the counseling process. All participants reported having awareness and knowledge of ototoxicity monitoring programmes and understood its importance. However, none of them reported the implementation of an ototoxicity monitoring programme in their facility.<br><br>Discussion<br><br>It is imperative to enhance the knowledge and understanding of the ototoxic nature of cancer treatment modalities among oncologists. It is crucial to raise awareness regarding the significance of ototoxicity monitoring programmes among all physicians involved in the treatment of patients with cancer for maximum impact. The barriers to the implementation of ototoxicity monitoring programmes in high- and low-income countries are similar. The models implemented in high-income countries can be adapted for use in low-middle income countries with suitable restructuring.
**Order of Authors:**	Varsha Shankar
	Satish Srinivas
	Raghu Nandhan
	Prasanna Kumar Saravanam

Powered by Editorial Manager® and ProduXion Manager® from Aries Systems Corporation

	Jayashree Seethapathy
**Additional Information:**	
**Question**	**Response**
**Financial Disclosure**<br><br>Enter a financial disclosure statement that describes the sources of funding for the work included in this submission. Review the submission guidelines for detailed requirements. View published research articles from *PLOS ONE* for specific examples.<br><br>This statement is required for submission and **will appear in the published article** if the submission is accepted. Please make sure it is accurate.<br><br>**Funded studies**<br>Enter a statement with the following details:<br>• Initials of the authors who received each award<br>• Grant numbers awarded to each author<br>• The full name of each funder<br>• URL of each funder website<br>• Did the sponsors or funders play any role in the study design, data collection and analysis, decision to publish, or preparation of the manuscript?<br><br>Did you receive funding for this work?	The author(s) received no specific funding for this work.
**Competing Interests**<br><br>Use the instructions below to enter a competing interest statement for this submission. On behalf of all authors, disclose any competing interests that could be perceived to bias this work—acknowledging all financial support and any other relevant financial or non-financial competing interests.<br><br>This statement is required for submission and **will appear in the published article** if the submission is accepted. Please make sure it is accurate and that any funding	The authors have declared that no competing interests exist.

Powered by Editorial Manager® and ProduXion Manager® from Aries Systems Corporation

sources listed in your Funding Information later in the submission form are also declared in your Financial Disclosure statement.<br><br>View published research articles from *PLOS ONE* for specific examples.<br><br>**NO authors have competing interests**<br><br>Enter: *The authors have declared that no competing interests exist.*<br><br>**Authors with competing interests**<br><br>Enter competing interest details beginning with this statement:<br><br>*I have read the journal's policy and the authors of this manuscript have the following competing interests: [insert competing interests here]*<br><br>* typeset	
**Ethics Statement**<br><br>Enter an ethics statement for this submission. This statement is required if the study involved:<br><br>• Human participants<br>• Human specimens or tissue<br>• Vertebrate animals or cephalopods<br>• Vertebrate embryos or tissues<br>• Field research<br><br>Write "N/A" if the submission does not require an ethics statement.<br><br>General guidance is provided below. Consult the submission guidelines for detailed instructions. **Make sure that all information entered here is included in the Methods section of the manuscript.**	This study was approved by the Institutional Ethics Committee of Sri Ramachandra Institute of Higher Education and Research (Deemed to be University) - (Reference no. IEC-NI/22/JUL/83/81). Verbal informed consent was obtained before the interviews, and it was audio-recorded.

**Format for specific study types**<br><br>**Human Subject Research (involving human participants and/or tissue)**<br>• Give the name of the institutional review board or ethics committee that approved the study<br>• Include the approval number and/or a statement indicating approval of this research<br>• Indicate the form of consent obtained (written/oral) or the reason that consent was not obtained (e.g. the data were analyzed anonymously)<br><br>**Animal Research (involving vertebrate animals, embryos or tissues)**<br>• Provide the name of the Institutional Animal Care and Use Committee (IACUC) or other relevant ethics board that reviewed the study protocol, and indicate whether they approved this research or granted a formal waiver of ethical approval<br>• Include an approval number if one was obtained<br>• If the study involved *non-human primates*, add *additional details* about animal welfare and steps taken to ameliorate suffering<br>• If anesthesia, euthanasia, or any kind of animal sacrifice is part of the study, include briefly which substances and/or methods were applied<br><br>**Field Research**<br><br>Include the following details if this study involves the collection of plant, animal, or other materials from a natural setting:<br>• Field permit number<br>• Name of the institution or relevant body that granted permission	
**Data Availability**<br><br>Authors are required to make all data underlying the findings described fully available, without restriction, and from the time of publication. PLOS allows rare exceptions to address legal and ethical concerns. See the PLOS Data Policy and FAQ for detailed information.	No - some restrictions will apply

A Data Availability Statement describing where the data can be found is required at submission. Your answers to this question constitute the Data Availability Statement and **will be published in the article**, if accepted.<br><br>**Important:** Stating 'data available on request from the author' is not sufficient. If your data are only available upon request, select 'No' for the first question and explain your exceptional situation in the text box.<br><br>Do the authors confirm that all data underlying the findings described in their manuscript are fully available without restriction?	
**Describe where the data may be found in full sentences. If you are copying our sample text, replace any instances of XXX with the appropriate details.**<br><br>• If the data are **held or will be held in a public repository**, include URLs, accession numbers or DOIs. If this information will only be available after acceptance, indicate this by ticking the box below. For example: *All XXX files are available from the XXX database (accession number(s) XXX, XXX.)*.<br>• If the data are all contained **within the manuscript and/or Supporting Information files**, enter the following: *All relevant data are within the manuscript and its Supporting Information files.*<br>• If neither of these applies but you are able to provide **details of access elsewhere**, with or without limitations, please do so. For example:<br><br>*Data cannot be shared publicly because of [XXX]. Data are available from the XXX Institutional Data Access / Ethics Committee (contact via XXX) for researchers who meet the criteria for access to confidential data.*<br><br>*The data underlying the results presented in the study are available from (include the name of the third party*	All relevant data is within the manuscript and its supporting information files.

and contact information or URL).<br>• This text is appropriate if the data are owned by a third party and authors do not have permission to share the data.<br><br>* typeset	
Additional data availability information:	

Manuscript Click here to access/download;Manuscript;Manuscript final draft.docx

Current practices and perspectives of oncologists regarding ototoxicity monitoring in individuals with head and neck cancers undergoing radiation therapy and chemoradiotherapy: A qualitative study in a South Indian district

Short title: Oncologist's perspectives of ototoxicity monitoring

Varsha Shankar[1], Jayashree Seethapathy[1*], Satish Srinivas[2&], Raghu Nandhan[3&], Prasanna Kumar Saravanam[4&]

[1]Department of Audiology, Sri Ramachandra Faculty of Audiology and Speech Language Pathology, Sri Ramachandra Institute of Higher Education and Research (Deemed to be University), Chennai, Tamil Nadu, India

[2]Department of Radiation Oncology, Sri Ramachandra Institute of Higher Education and Research (Deemed to be University), Chennai, Tamil Nadu, India

[3]Department of ENT – Head & Neck Surgery, Madras ENT Research Foundation, Chennai, Tamil Nadu, India

[4]Department of ENT, Head and Neck Surgery, Sri Ramachandra Institute of Higher Education and Research (Deemed to be University), Chennai, Tamil Nadu, India

***Corresponding author:**

Dr. Jayashree Seethapathy

jayashree.s@sriramachandra.edu.in (JS)

[&]These authors contributed equally to this work.

Abstract

Aim: The current study aims to explore the oncologist's awareness and perspectives of ototoxicity and ototoxicity monitoring for individuals with head and neck cancer in a South Indian district, using qualitative semi-structured interviews.

Method: The study utilized the COREQ Checklist to guide the method of the current study. A conceptual framework was developed for the formulation of the interview guides. Three medical oncologists and six radiation oncologists from tertiary centres pioneering in cancer care participated in the study. The interviews were audio recorded and transcribed verbatim. Thematic analysis was carried out using a hybrid inductive-deductive approach to present the findings under the respective over-arching themes.

Results: All oncologists are aware of the ototoxic effects caused by radiation therapy and chemoradiotherapy. It was consistently reported that the severity of the hearing loss was not substantial enough to warrant significant concern. Ototoxicity is not emphasized during the counseling process. All participants reported having awareness and knowledge of ototoxicity monitoring programmes and understood its importance. However, none of them reported the implementation of an ototoxicity monitoring programme in their facility.

Discussion: It is imperative to enhance the knowledge and understanding of the ototoxic nature of cancer treatment modalities among oncologists. It is crucial to raise awareness regarding the significance of ototoxicity monitoring programmes among all physicians involved in the treatment of patients with cancer for maximum impact. The barriers to the implementation of ototoxicity monitoring programmes in high- and low-income countries are similar. The models implemented in high-income countries can be adapted for use in low-middle income countries with suitable restructuring.

Introduction

In India, head and neck cancers (HNC) is one of the most common malignancies reported in both males and females [1]. One in 33 males and one in 107 females are at risk of developing the disease in their lifetime [2]. Treatment for HNCs has evolved over the past decade and today multi-modality therapy consisting of radiation therapy (RT), along with surgery, chemotherapy and immunotherapy, cure an increasing percentage of patients with locally advanced cancers. While conservative surgery or RT are equally effective in early stages I and II of HNC, locally advanced stages III and IV require radical resection coupled with reconstruction and post-operative RT or concurrent chemoradiotherapy (CCRT) with platinum compounds to effect cure [1]. Patients with metastatic cancer are palliated with a combination of short course RT and systemic therapy for symptom relief and maintain or improve quality of life [3].

Surgical (SO), Medical (MO) and radiation oncologists (RO) collaborate to provide treatment for patients with HNC. The treatment depends upon stage, site and complexity of the disease [4]. While survival rates and life expectancy have increased post-treatment, these treatment options might negatively impact quality of life due to their effects on swallowing, speech, hearing, and overall psychological well-being of the patient [5]. Thus, the treatment process also involves an integrated multidisciplinary care approach with periodic referrals to dentists, clinical psychologist and speech and swallow therapists to manage side effects such as mucositis, odynophagia and dysphagia [6]. These referrals are periodic and streamlined, due to the impact of these side effects on the prognosis of the disease [6].

RT and CRT have been documented to cause ototoxicity, as they can impact the structures of the inner ear [7]. Ototoxicity is the cellular degeneration of cochlear and/or vestibular tissues that leads to its functional deterioration due to the use of certain therapeutic agents [8]. The incidence of sensorineural hearing loss (SNHL) after RT and CRT ranges from 0% to 43% and 17% to 88%, respectively [9]. In addition to the existing burden of disease, hearing loss can lead to communication difficulties and social disengagement. It can lead to challenges in cognition, child development, and academic achievement among children. In adults, RT/CRT can result in poor balance which interferes with walking and increases the risk of falling. Ototoxicity

is also linked to depression and anxiety [10]. Thus, hearing loss due to RT/CRT is an important "quality-of-life" issue which should not be overlooked.

Ototoxicity monitoring (OM) tracks hearing and vestibular changes over time, and alerts the consulting physician when changes are detected early. This can result in opting for alternative treatment protocols, possibly with less ototoxic medications [11]. There are several OM guidelines for patients receiving RT or CRT [12, 13]. According to ASHA and AAA guidelines, OM needs to be carried out at baseline, during treatment and at 1 months, 3 months and 6 months post-treatment [12, 13]. A coordinated referral from oncologists to the audiologist is essential to optimise OM. A few international studies explored the perspectives and practices of oncologists regarding referrals to audiology for OM from South Africa and USA.

The perspectives and practices of healthcare professionals regarding cisplatin-associated ototoxicity in individuals with cancer was described in a South African hospital-based study [14]. The study findings indicated that OM was not included in the chemotherapy (CT) protocols and no ototoxicity monitoring programme (OMP) was implemented. All audiologists reported that the oncology department does not refer patients for routine OM. Similarly, a written survey/survey-based interview was used to report the perspectives of four physicians on OMP provision in the United States [15]. The interviewees reported that physicians' approaches to OM varied, with only some ensuring routine referrals to audiology while others relied on patient reported symptoms and self-referrals. The referral processes were based on the respective oncologist's perceptions of the expected risk of ototoxicity.

In recent years, efforts in India have increased to establish regional cancer centres in rural areas, and upgrade medical institutions with oncology department to address the populations' cancer care needs. It is worth noting that there are several tertiary cancer centres with state-of-the-art diagnostic workup and treatment protocols that improve the detection and treatment of various types of cancers [16]. However, these protocols are yet to meet international standards; the need of the hour is to focus on improving the quality-of-life of cancer patients.

Despite the prevalence of HNC in the Indian population, there is limited evidence documenting the quality-of-life post-treatment in Indian literature [17]. There is a lacuna pertaining to literature on periodic OM in

India [14] and ototoxicity research is still in its early stages [11]. There are no studies reporting the practises and awareness of ototoxicity among oncologists treating cancer patients in the South Indian context.

Thus, the current study aims to explore the oncologist's awareness and perspectives of ototoxicity and OM for individuals with HNC in a South Indian district, using qualitative SSIs. The specific objectives of the study are (a) to understand the current practices of oncologists in the treatment of individuals with HNC, (b) to understand the oncologist's perspectives regarding ototoxicity and OMPs for individuals with HNC undergoing RT and CRT.

Materials and Methods

This study was approved by the Sri Ramachandra Institutional Ethics Committee (Reference no. IEC-NI/22/JUL/83/81). The study utilized the COnsolidated criteria for REporting Qualitative research (COREQ) Checklist [18] to guide the method (S1 Appendix 1).

Conceptual Framework

A conceptual framework (Fig 1) was developed based on the constructs of the Health Belief Model [19] and a review of the literature. It was utilized for the formulation of the interview guide. The interview guide (S2 Appendix 2) was pilot tested on two participants and was modified based on the participant's feedback. The themes have been operationally defined in the context of the current study (Table 1).

Fig 1. Theoretical framework developed and used for the current study.

Table 1. Operational definition of themes used in the theoretical framework.

S.No	Theme	Definition
1.	Current Practices	Current practices used by Oncologists in the treatment of individuals with Head and Neck Cancer – treatment options, techniques used, counselling, side effects, management of side effects, audiological monitoring etc.
2.	Perceived susceptibility	Oncologist's belief about an individual with HNC at risk of developing ototoxicity
3.	Perceived barriers	Oncologist's belief about the challenges in implementing an OMP
4.	Cues to action	Oncologist's suggestions and strategies for the future implementation of OMPs
5.	Perceived Utility	Oncologist's perceptions regarding confidence to take action, perceptions about the potential success and impact of the OMP

Study site and sampling

Medical oncologists (MOs) and radiation oncologists (ROs) from tertiary care centres with a large number of patients with HNCs were approached to participate in the study. It was ensured that all participants had at least one year of experience, so that sufficient information and perspectives related to ototoxicity could be obtained [20]. Some of the participants referred the interviewers to other MOs and ROs; thus, the participants were selected using a snowball sampling method [21]. Overall, approximately 20 MOs and ROs from these centres/institutions were approached in-person or through known contacts for participation in the study.

Participants were approached through phone calls to describe the study, obtain consent, schedule an appointment and conduct the SSI. No relationship was established prior to the commencement of the study and participant knowledge of the interviewers was minimal. Participants who consented to participate were included in the study and the background and aim of the study were explained in the informed consent. Interviewer's interests in the topic and the reason for conducting the SSIs was clarified to the participant prior to obtaining consent from all the participants. Verbal informed consent was obtained before the interviews and it was audio-recorded.

Interview details

The interviews were conducted at the tertiary care centres. Two female authors (VS and JS) conducted the SSIs. Both authors were trained in qualitative research and have prior experience with conducting SSIs. Apart from the researchers and participants, no one else was present during the interviews to ensure that confidentiality was maintained. The interviewers used questions and prompts from the interview guide to conduct the SSIs which lasted approximately 30 – 45 minutes. Repeat interviews were not carried out. The interviews were audio recorded and field notes were maintained. Later, the audio recordings of the interviews were transcribed verbatim and the transcripts were verified by the participants. As soon as redundancy was detected in the acquired data, data saturation was attained [22]. Thus, six ROs and three MOs participated in the interviews.

Analysis

Thematic analysis was carried out using a hybrid inductive-deductive approach [23] with the QDA Miner Lite software. Two authors (JS and VS) coded the data and provided a description of the coding tree, independently. The authors became familiarized with the transcripts and added new codes to the current codebook based on inferences drawn from the interviews. Coding inconsistencies and differences were discussed and rectified. The remaining transcripts were coded using the refined codebook and new codes were assigned to unique and significant information discovered during this process.

The codes were organized according to similarities and the frequency of occurrences, which assisted in classification. These categories were analysed to identify significant patterns in the data and the findings were grouped under the respective over-arching themes. This was followed by an assessment of the themes with regard to their applicability and credibility. Certain themes were combined during this process in order to present the findings in a more streamlined manner.

Participant quotations have been presented to substantiate the themes and findings. The quotes have been identified using specific identifiers. Both major and minor themes have been described along with emphasis on unique findings. The findings were sent to the participants for feedback and “no objections for publication”.

Results

Nine participants provided consent to participate in the interviews. The participant characteristics such as profession, years of experience, gender and participant setting are described in table 2.

Table 2: Participant characteristics - profession, years of experience, gender, setting.

S.No	Profession	Code	Years of experience	Gender	Setting
1.	Radiation Oncologist	RO1	2.5 years	Male	Private quaternary care centre
2.	Medical Oncologist	MO1	30 years	Male	Private quaternary care centre
3.	Radiation Oncologist	RO2	25 years	Male	Public charitable comprehensive cancer centre
4.	Radiation Oncologist	RO3	1.5 years	Female	Private quaternary care centre
5.	Medical Oncologist	MO2	3 years	Female	Public charitable comprehensive cancer centre
6.	Medical Oncologist	MO3	3 years	Male	Private quaternary care centre
7.	Radiation Oncologist	RO4	8 years	Male	Private quaternary care centre
8.	Radiation Oncologist	RO5	23 years	Male	Private multi-speciality quaternary care hospital
9.	Radiation Oncologist	RO6	11 years	Male	Private multi-speciality quaternary care hospital

The results of the current study are explained and presented under the following themes identified in the conceptual framework:

(a) Current practices for the treatment and management of HNC
- Medical Treatment for HNC
- Counselling
- Side effects of treatment and its management

(b) Ototoxicity
- Ear and hearing related side effects
- Perceived susceptibility to ototoxicity

(c) Ototoxicity Monitoring
- Current practices of OM and challenges

- Perceived barriers
- Cues to action
- Perceived efficacy

The quotes are described using the respective identifier codes in S3 Appendix 3

Theme 1 - Current practices for the treatment and management of HNC:

Medical treatment for HNC

All interviewees stated that the treatment options for HNC are determined by the type, stage, and other special considerations. The tumor staging is commonly categorized as early stage, locally advanced stage, and metastatic or recurrent stage. Early-stage tumors are treated with a single modality, which is either surgery or RT. Surgery and/or CCRT are used to treat locally advanced malignancies. The metastatic cancers are treated using chemotherapy alone (refer to quote 1). CCRT was reported to be the most often utilized treatment plan for HNC (refer to quote 2). According to one interviewee, CCRT is used as a treatment modality as chemotherapy acts as a radiosensitizer for enhancing the effects of RT (refer to quote 3).

Cisplatin is the most often used platinum agent to treat HNC, according to all participants, and it is typically administered in two regimens: 100 mg/m^2 once in three weeks or 40 mg/m^2 every week for five to six weeks. The treatment regimen is chosen based on several factors, such as the patient's condition, co-morbidities, and toxicity profile (refer to quote 4).

With the advent of modern linear accelerators, many RT techniques such as Intensity Modulated Radio-Therapy (IMRT), Image Guided Radio-Therapy (IGRT), Volumetric Modulated Arc Therapy (VMAT) are available. In terms of imaging protocol, IGRT differs from IMRT in that pre-treatment image verification occurs with inbuilt on-board kilovolt (kV) cone beam CT imaging as opposed to the megavolt (MV) X-ray imaging used in IMRT. Because it avoids/limits dosage to all "organs at risk," IMRT is the treatment technique of choice for patients with HNC. As a result, it has fewer side effects and toxicities (refer to quote 5).

An MO and RO reported that in certain conditions, neo-adjuvant treatment is administered prior to surgical removal of the tumor. This is typically performed in situations when specific attention is needed, such as when the preservation of the larynx is a priority or when there is trismus (refer to quotes 6 and 7).

The treatment technique is determined by the tumor characteristics (site, stage and volume of the disease) and the patient characteristics (age and general condition of the patient). According to one MO, the risk of toxicity in elderly patients is assessed using a scoring system which aids the physician in the drug selection process (refer to quote 8). In some instances, the choice of technique may be influenced by the patient's affordability (refer to quote 9). One MO specified that treatment is decided in multidisciplinary tumour board discussions (refer to quote 10).

Counselling

Both MOs and ROs agreed that counseling is an essential component of the treatment process and is contingent on several factors, such as the site, stage, and prognosis of the disease. Treatment options, duration, and other relevant aspects are highlighted during the counseling session. All participants reported that counseling begins upon diagnosis of the disease and continues periodically during the course of treatment, accompanied by a timely review of the patient's status (refer to quote 11). This reportedly ensured that all patient queries were addressed periodically. In addition, one MO emphasized the importance of counseling patients regarding stopping the use of tobacco before treatment is initiated (refer to quote 12).

One RO reported that the counseling process in India differs from that of western countries. The patient is counseled only briefly about the disease and treatment. However, it is the caregivers of the patients who are given a detailed explanation about the prognosis and severity of the disease (refer to quote 13). He also stated that the patient should be aware of potential side effects. This awareness can help in patient compliance and fear reduction, which influences the patient's willingness to continue treatment. According to the participant, if proper counseling is provided, patients cooperate better with physicians and are better equipped to manage their symptoms (refer to quote 14).

When asked about counseling regarding ototoxicity, one MO stated that ototoxicity doesn't receive significant emphasis. He stated that ototoxicity often impacts only the higher frequencies, and only when the cumulative dosage surpasses the thresholds will it influence the lower frequencies. Therefore, he did not perceive the necessity to emphasize ototoxicity during the counseling (refer to quote 15).

Side effects of treatment of HNC and its management

The interview participants reported that RT and CRT can result in several side effects. The CT-associated side effects include renal toxicity, chemotherapy-induced nausea and vomiting (CINV), diarrhea, low blood count, nutritional deficiencies, and hyponatremia (refer to quote 16).

ROs reported that patients often complained of xerostomia, mucositis, dysphagia, odynophagia, dental problems, and osteo-radio-necrosis. They stated that RT compromised the physiology of the structures on the affected side, which were usually the sites of irradiation. Since midline structures would be treated with wider fields of irradiation, bilateral structures would be affected. These side effects were reported to be acute and reversible. Nutrition was reported to be a crucial factor for effective treatment outcomes, and ROs prioritize maintaining optimal food intake throughout the treatment (refer to quotes 17, 18 and 19).

MOs and ROs indicated that management of side effects involves the use of a grading scale to assess the severity through regular monitoring. If the side effects are classified as grade three or above, treatment is paused and symptomatic management is implemented (refer to quotes 20 and 21). None of the interview participants reported the grading of ototoxicity. Symptomatic management of the side effects includes prescribing a combination of medications for CINV, topical applications for the management of mucositis, mouthwashes to prevent oral infections, analgesics for pain, etc (refer to quotes 22 and 23).

For proactive management of these side effects, ROs stated that all patients with HNC undergoing RT are referred to dentists prior to treatment initiation. Patients are referred to speech and swallow therapists for rehabilitation of speech, swallow, and voice disorders secondary to treatment (refer to quote 24). Only one RO reported that patients with nasopharyngeal tumors are referred to an audiologist and an ophthalmologist

due to the near proximity of the tumor site to the optic apparatus and cochlea. Baseline evaluation measures are completed, and follow-up measures may be performed to assess any changes (refer to quote 25).

Theme 2 - Ototoxicity

Ear and hearing related side effects

The interview participants were probed regarding the ear and hearing-related side effects. One MO opined that the disease

can itself cause changes in hearing, and cisplatin as a therapeutic agent can exacerbate these changes (refer to quote 26). Another MO stated that hearing loss was a late toxicity and is usually reported by patients towards the end of treatment or during the follow-up (refer to quote 27).

Among ROs, there were conflicting reports of ear and hearing-related side effect. One RO reported that hearing loss was a late side effect (refer to quote 28). Two other ROs reported that ear-related complaints are not late side effects and may be observed in the acute phase (refer to quotes 29 and 30). However, these were reported to be temporary and included serous otitis media or aural fullness.

When probed regarding the management of these ear and hearing-related complaints, the majority of the oncologists reported that these symptoms were treated symptomatically. Common management practices by ROs included obtaining an ENT opinion, and prescribing otic analgesics and antibiotics. These management strategies alleviated the symptoms (refer to quote 31). Only one MO emphasized the significance of baseline audiological evaluation as a management option. He believed that documenting the baseline hearing thresholds and monitoring the ototoxic effects was beneficial. He felt that this could influence the modification of the treatment and dose of cisplatin (refer to quote 32).

Perceived susceptibility to ototoxicity

The oncologist’s perceptions regarding susceptibility to ototoxicity were explored. ROs believed that ototoxicity caused by RT was rare because only a limited dose of radiation reaches the cochlea; cochlear

contouring is performed during the treatment planning phase (refer to quotes 33 and 34). Hearing-related complaints were predominantly associated with the chemotherapeutic agent, which is ototoxic in nature, as opposed to RT.

One MO expressed the opinion that, given the patient's life-threatening illness and side effects, addressing hearing loss may not be a top priority (refer to quote 35). When inquired about the potential impact of an ototoxicity diagnosis on the physician's treatment decision, one RO reported that it would impact the choice of chemotherapeutic regimen. He stated that the treatment plan of the RO is not considerably affected by ototoxicity, as ROs already implement technical precautions to minimize the dose to the cochlea from the time of treatment initiation (refer to quote 36).

A RO stated that hearing loss seldom receives adequate consideration for two main reasons: oncologists may mistakenly believe that the unaffected ear may compensate for the hearing loss. Alternatively, they may also believe that hearing loss can always be effectively managed with rehabilitative measures (refer to quote 37).

Theme 3 - Ototoxicity monitoring programmes

The participants' awareness and perceptions of OMPs were explored during the interviews. This included questions regarding the current status of OMPs in their center and other centers across Tamil Nadu. All participants were aware of OMPs, possessed minimal knowledge of OMPs and understood its significance. However, none of them reported the implementation of an OMP in their center. They were unaware of any other centers implementing routine OMPs (refer to quote 38).

All participants stated that there is scope for improvement in the evidence-base on ototoxicity in the country and felt that there is a need to investigate ototoxicity, particularly in rural settings (refer to quote 39). An RO admitted that he was unaware of the radiation tolerance dose and the contraindications for RT in patients with ear and hearing-related issues (refer to quote 40).

Current practices of OM and challenges

The interview participants were asked about their current practices of OM. An MO and RO stated that while they are aware of the importance of routine OM, they have never referred any patient to an ENT specialist or an audiologist for audiological evaluation. They reported that case history regarding hearing is not obtained frequently (refer to quotes 41 and 42).

Another MO stated that hearing loss will be investigated only if the patient complains of it. Hearing loss often goes undetected as patients commonly exhibit proficient communication and interaction with oncologists, causing oncologists to never notice any auditory changes. Furthermore, she believed that oncologists would be capable of monitoring for ototoxicity if they had previous clinical experience (refer to quote 43). Similarly, an RO reported that baseline audiological evaluations are only performed when hearing loss is suspected. This referral is subjective and is based on the oncologist's perceptions of the patient's hearing status during a conversation. Baseline audiograms were only used to ensure that the hearing loss was not caused by the treatment (refer to quote 44).

Only one RO reported performing baseline audiological evaluations on nasopharyngeal cancer patients who complained of ear and hearing problems. These patients were referred for an ENT opinion three months after their treatment, but follow-up audiological measures were not carried out. Routine OM is not performed for patients with other types of HNC (refer to quote 45).

According to the RO of a public charitable comprehensive cancer center, the medical oncology department uses a scale or a scoring system to screen for hearing loss and determine the appropriate drug dose. Baseline audiograms are requested in cases of a pre-existing ear condition or hearing loss (refer to quote 46). However, the MO at the same centre indicated that only a history is recorded and baseline audiograms are not recommended for all patients (refer to quote 47). Another MO reported that patients are often monitored for vomiting, myelosuppression, tiredness, pain, etc., but not for ototoxicity. Baseline measures of echocardiogram and other tests are carried out; however, baseline audiometry is not performed for any patient due to logistic constraints (refer to quote 48).

The various challenges experienced by oncologists in undertaking OM were explored. According to one MO, some of these challenges include higher costs associated with audiological evaluation and increased testing

duration (refer to quote 49). Due to the lack of an in-house audiology department, the oncologist faced challenges in recommending patients for audiological evaluations. From the patient's perspective, undergoing audiological examinations was difficult due to a lack of financial and/or family support (refer to quote 50). Another MO stated that patients may perceive a delay in their treatment due to the audiological evaluation. Audiological evaluation may not be a priority in comparison to the discomfort and pain they are feeling; they may not be healthy or comfortable enough to wait and undertake a comprehensive audiological evaluation (refer to quote 51).

When patients were referred for audiological evaluation, there were ambiguous complaints of hearing loss by patients due to their lack of understanding. In addition, inaccuracies and ambiguity in the diagnosis of hearing loss in the audiological reports were a challenge (refer to quote 52). An RO also reported that patients do not complain of hearing loss after treatment due to a lack of awareness or ignorance. Other symptoms and concerns take precedence over hearing loss, which may explain why oncologists do not monitor hearing on a frequent basis (refer to quote 53). Thus, ototoxicity has been limited to literary awareness and knowledge rather than clinical translation.

Perceived barriers

Oncologists' perspectives on the barriers to OMP implementation in the future were explored. According to one RO, stakeholders may exhibit reluctance to accept this program in its early phases, but this challenge can be addressed (refer to quote 54). In addition, another RO expressed the difficulty of persuading patients to undergo regular audiological assessments, as they tend to prioritize initiating their cancer treatment promptly. He felt that it would be a challenge to convince them to undergo multidisciplinary consultations with dentists, ophthalmologists, and other specialists (refer to quote 55). Scheduling a mutually convenient time for audiological evaluations was also reported to be a challenge (refer to quote 56).

The organization may perceive the establishment of an audiology clinic and the procurement of equipment as a costly undertaking. The OMP has to be cost-effective to ensure organizational acceptance and sustainability (refer to quote 57). Finally, one RO stated that he did not anticipate any major challenges during the

implementation phase. He felt that challenges and solutions can only be identified when implementation begins (refer to quote 58).

Cues to action

All interviewees were asked for their suggestions and strategies to implement the OMP. One RO reported that an OMP can be implemented by utilizing existing frameworks and evidence found in the literature. He believed that this would allow for the prediction of the cost-benefit analysis. He also recommended collaborating with the institute's department of speech, language, and hearing sciences (refer to quote 59).

Interview participants were asked about the organizational support required for OMP implementation. Adequate infrastructure and a dedicated audiology unit in the oncology department will be beneficial for counseling and the referral process. The patient will have the convenience of an in-house audiologist, eliminating the need for travel and ensuring greater accessibility (refer to quotes 60 and 61). Alternatively, an MO and RO suggested arranging regular visits from an audiologist to the oncology unit to perform routine OM. They believed that this system would promote follow-up and facilitate effective collaboration between the audiologist and the oncologist (refer to quotes 62 and 63). Another MO proposed aligning the audiology clinic's operating hours with those of the cancer clinics to streamline referrals (refer to quote 64). When asked about the need for human resources during the implementation process, a RO from a private quaternary care center stated that they had sufficient manpower. However, he perceived that the entire referral process requires optimization (refer to quote 65).

The interview participants were asked about the multi-disciplinary role-players required in an OMP. A RO stated that both the oncologist and the audiologist should be involved throughout the entire counseling process, with the oncologist acting as the primary point of contact for the patient. The oncologist should support and enable the audiologist's involvement to ensure the patient's ease. Two other ROs agreed that the multidisciplinary team should include ENT specialists, clinical psychologists, and pain and palliative care specialists (refer to quotes 66, 67, and 68). According to a MO, the audiologist's primary responsibility would be to plan the management and rehabilitation of hearing loss (refer to quote 69).

The oncologists were asked about strategies to increase patient acceptance of an OMP. An experienced RO suggested that patients might find OMPs to be useful if they were cost-effective and had a noticeable impact on their daily lives. It should be accessible and affordable (refer to quotes 70 and 71). An RO gave an interesting suggestion to consolidate the billing of allied health care services into a single package; the patient will not feel overburdened by the number of diagnostic evaluations they have to undergo and will believe that he is receiving valuable, multi-disciplinary care for his disease (refer to quote 72). Another RO believed that patients would quickly accept the new program provided they were adequately counseled and understood that routine audiological evaluations were being conducted for their own benefit (refer to quote 73). Likewise, the program's acceptance can be enhanced by prioritizing ototoxicity from the beginning and ensuring that the patient feels empowered in the decision-making process (refer to quote 74). Such a patient-centered approach will not only increase awareness of hearing loss but will also encourage patients to report any complaints of hearing loss to their team of specialists as soon as they begin experiencing challenges (refer to quote 75).

The interview participants were asked about the ideal timeline for conducting audiological evaluations. Both MOs and ROs agreed that audiological evaluations should be conducted before the initiation of cancer treatment and periodically throughout the treatment process. Subsequent assessments should be conducted at three months and six months post-treatment to account for late treatment effects (refer to quotes 76 and 77).

Regarding patient counseling, a RO opined that the responsibility of ototoxicity counseling should be initially assumed by the oncologist and thereafter by the audiologist. This would eliminate confusion and miscommunication between the team members. Engaging the services of a qualified psychologist during the treatment process can provide patients with a sense of support and assistance during their treatment (refer to quotes 78 and 79).

Perceived utility

Oncologists' perceptions regarding the possible impact of the OMP, it's potential success and their confidence to take action were explored during the SSIs. ROs believed that OMPs would provide reliable information regarding the patient's hearing status, aid in treatment planning, and also improve the overall quality of service

delivery (refer to quotes 80 and 81). Routine audiological evaluation and documentation of the hearing status could assist oncologists in reporting that the patient's hearing loss occurred prior to the treatment and was not caused by the treatment itself. This can help avoid any medico-legal issues in the future (refer to quote 82). A MO stated that OM will allow for identification of hearing loss in pediatric patients, which can significantly influence their overall development (refer to quote 83).

Another RO noted that the OMP should be disseminated to all healthcare professionals involved in cancer treatment to maximize its effectiveness (refer to quote 84). According to an RO, routine OM will instill confidence in the stakeholders to continue it in a sustained manner (refer to quote 85). In addition, all oncologists expressed their willingness to collaborate with an audiologist for the OMP implementation. Almost all oncologists responded positively when asked if they would be willing to change their practice to include an OMP because an audiologist would be in charge of it. A RO added that context-specific challenges will arise only during the implementation; he felt that the OMP should be introduced with a few patients and modifications should be made during the implementation (refer to quote 86).

Discussion

The current study used a qualitative approach to ascertain the awareness and perspectives of ototoxicity and OM among oncologists treating individuals with HNC. Their perceptions regarding the barriers and strategies for OM were also explored.

The study findings revealed that all oncologists are aware of the ototoxic effects caused by RT and CT. They reported that hearing loss is rarely observed and a case history regarding the hearing status is also not asked frequently. All oncologists reported that perceivable ototoxicity may be a late toxicity that patients typically report towards the end of treatment or during follow-up. ROs had different opinions regarding the acuteness of the ototoxicity. There was a prevailing belief that ear and hearing-related complaints due to RT were exceedingly uncommon. It was constantly reported that the severity of the hearing loss was not substantial enough to warrant significant concern. Ototoxicity is not emphasized during the counseling process, as well.

It was interesting to note that the common perception was that hearing was not considered a priority when the patient was experiencing other life-threatening complaints.

These perceptions could be due to various factors. There is a preconceived notion that only a restricted dose of radiation reaches the cochlea, thereby protecting it from the effects of radiation. ROs also felt that the chemotherapeutic agent was more ototoxic compared to RT. Moreover, it is widely considered that the higher frequencies are impacted initially, while the lower frequencies are affected later when the cumulative dose exceeds the prescribed limits. Oncologists may argue that if one cochlea is affected during the treatment, the contralateral cochlea has the potential to compensate for the loss of the affected sensory organ. Alternatively, they may also believe that hearing loss can always be effectively addressed with rehabilitative options. Not only is hearing loss nonfatal, but in most cases, patients respond and communicate well with the treating physician. Thus, the treating physician does not suspect hearing loss. Typically, patients do not report any hearing problems and are unaware that their hearing loss was a result of the treatment.

Contrary to the beliefs of oncologists, numerous studies have reported that radiation-induced hearing loss is one of the most common side effects of radiation in individuals with HNC [24]. A systematic review reported that the incidence of RT-induced SNHL ranged from 0% to 43% [9]. Hence, it is imperative to enhance the knowledge and understanding of the ototoxic nature of cancer treatment modalities among oncologists. There is a significant need to update oncologists regarding the incidence and prevalence of ototoxic hearing loss induced by RT and CT. This finding aligns with previous studies [14]; there is a need to increase awareness among nursing staff regarding the ototoxic nature of chemotherapeutic drugs. This would facilitate counseling and empower patients to promptly inform their treating physicians of any changes in their hearing status. Patients need to be informed of the early symptoms of ototoxicity to ensure its early identification and management.

All participants demonstrated awareness and knowledge of OMPs and understood its importance. However, none of them reported the implementation of an OMP in their facility. In addition, they were not aware of any other centers implementing OMPs on a regular basis. Although oncologists acknowledge the importance of OM, they seldom make referrals to an ENT specialist or an audiologist. The referrals are subjective and are

based on the oncologist's perceptions of the patient's hearing status when communicating with them. Thus, ototoxicity has been restricted to literary awareness and knowledge without clinical translation [11].

It is crucial to raise awareness regarding the significance of OMP among all physicians involved in the treatment of patients with cancer for maximum impact [25]. The findings of the current study are similar to those reported previously [15]. The authors reported the use of varied referral systems, including routine referrals to audiology and relying on patient self-referral. Oncologist and other stakeholder input is needed to strengthen monitoring schedules and streamline OMP care coordination with audiology.

Both oncologists and audiologists need to be involved in the entire counseling process, with the oncologist serving as the primary contact for the patient [25]. The oncologist should facilitate the audiologist's involvement for the patient's ease. Prioritizing ototoxicity right from the beginning and ensuring that the patient feels autonomous in the decision-making process will facilitate treatment acceptance. Implementing a patient-centric approach will not only raise awareness of hearing loss but also motivate patients to proactively communicate any hearing-related concerns to their team of specialists.

The SSIs explored the various reasons for the dearth of OM practices. The challenges include increased cost associated with audiological evaluation, long testing times, patients lacking the physical well-being or comfort to endure the wait for their audiological evaluation, ambiguous complaints of hearing loss by patients due to their lack of understanding, and errors and ambiguity in the diagnosis of hearing loss in the audiological reports. Lack of an in-house audiology department for ease of referrals was reported to be a significant challenge. From the patient's end, lack of affordability and/ or family support were challenges to undergoing audiological evaluations. The major barriers anticipated by the oncologist in the future implementation of an OMP included (a) the initial cost to set up, (b) initial hesitation from stakeholders, (c) conflict in scheduling the audiological evaluation and the cancer treatment, and (d) difficulty convincing the patients of their hearing loss. The management must recognize that OMPs have a favorable cost-to-benefit ratio, as their approval is essential for the long-term sustainability of the OMP. These findings were similar to the barriers reported in other developed nations [26, 27]. Thus, the barriers to the implementation of OMPs in high- and low-income

countries are similar. The models implemented in high-income countries can be adapted for use in LMICs with suitable restructuring.

All interview participants were asked for their suggestions and strategies to sensitize and implement the OMP in a seamless manner. These suggestions included (a) adopting frameworks and evidence from existing literature for implementation; (b) establishing infrastructure to effectively address patient needs and provide counseling; (c) setting up protocols to streamline the referral process; and (d) having an in-house audiologist or audiology department to facilitate the interpretation of audiological reports and a seamless referral process. One interesting suggestion was to develop a regular schedule for an audiologist to visit the oncology ward for routine OM. All essential services for the treatment of HNC, including allied healthcare, can be invoiced as a package. This may ensure that the patient does not feel overwhelmed by the associated cost of the extensive evaluation and instead feels he is receiving valuable, multi-disciplinary care for his condition. Konrad-Martin et al. (2018) proposed the utilization of screening methods to facilitate monitoring and the incorporation of audiological management into standard care. The authors stated that adherence to guidelines might potentially be enhanced with the formal support of other stakeholders involved with OMPs. In addition, tele-audiology has proven to be an effective method for delivering OM [28].

Oncologists' perceptions regarding the confidence to take action and perceptions about the potential success and impact of the OMP were explored during the SSIs. ROs believed that OMPs would provide accurate results regarding the patient's hearing status, therefore aiding in treatment planning. It will also improve the overall quality of service delivery. Additionally, physicians might utilize it to document evidence of the patient's pre-existing hearing loss, which was not caused by the treatment. This enables clinicians to record the findings in order to mitigate potential medico-legal issues in the future. All oncologists expressed their willingness to include an audiologist in the establishment of an OMP.

The strength of the current study lies in its robust methodology. This study is among the first in South India to determine the perspectives, awareness, and knowledge of ototoxicity among oncologists treating individuals with HNC using a qualitative approach. The findings of the current study can influence the implementation of OMPs in the future. The [illegible]tations of the current study include a lack of a data triangulation method. Despite

several attempts to increase the sample size of the medical oncology population, obtaining consent proved to be a challenge.

Acknowledgements

We would like to thank all the oncologists for their participation in the SSIs.

References

[1] Dandekar M, Tuljapurkar V, Dhar H, et al. Head and neck cancers in India. *J Surg Oncol* 2017; 115: 555–563.

[2] Bagal S, Budukh A, Thakur JS, et al. Head and neck cancer burden in India: an analysis from published data of 37 population-based cancer registries. *Ecancermedicalscience*; 17. Epub ahead of print 1 September 2023. DOI: 10.3332/ecancer.2023.1603.

[3] Pulte D, Brenner H. Changes in Survival in Head and Neck Cancers in the Late 20th and Early 21st Century: A Period Analysis; Changes in Survival in Head and Neck Cancers in the Late 20th and Early 21st Century: A Period Analysis. *Oncologist* 2010; 15: 994–1001.

[4] Chambers MS, Garden AS, Kies MS, et al. Radiation-induced xerostomia in patients with head and neck cancer: Pathogenesis, impact on quality of life, and management. *Head and Neck* 2004; 26: 796–807.

[5] Hitchcock YJ, Tward JD, Szabo A, et al. Relative Contributions of Radiation and Cisplatin-Based Chemotherapy to Sensorineural Hearing Loss in Head-and-Neck Cancer Patients. *Int J Radiat Oncol Biol Phys* 2009; 73: 779–788.

[6] Townsend M, Kallogjeri D, Scott-Wittenborn N, et al. Multidisciplinary clinic management of head and neck cancer. In: *JAMA Otolaryngology - Head and Neck Surgery*. American Medical Association, 2017, pp. 1213–1219.

[7] Ganesan P, Schmiedge J, Manchaiah V, et al. Ototoxicity: A challenge in diagnosis and treatment. *J Audiol Otol* 2018; 22: 59–68.

[8] Arslan E, Orzan E, Santarelli R. Global problem of drug-induced hearing loss. *Ann N Y Acad Sci* 1999; 884: 1–14.

[9] Theunissen EAR, Bosma SCJ, Zuur CL, et al. Sensorineural hearing loss in patients with head and neck cancer after chemoradiotherapy and radiotherapy: A systematic review of the literature. *Head Neck* 2014; 36: 1391.

[10] Phillips OR, Baguley DM, Pearson SE, et al. The long-term impacts of hearing loss, tinnitus and poor balance on the quality of life of people living with and beyond cancer after platinum-based chemotherapy: a literature review. *Journal of Cancer Survivorship* 2023; 17: 40.

[11] Paken J, Govender CD, Pillay M, et al. Cisplatin-Associated Ototoxicity: A Review for the Health Professional. *J Toxicol*; 2016. Epub ahead of print 2016. DOI: 10.1155/2016/1809394.

[12] ASHA. Audiologic Management of Individuals Receiving Cochleotoxic Drug Therapy. *Med Pediatr Oncol* 1994; 1–17.

[13] AAA. American Academy of Audiology Position Statement and Clinical Practice Guidelines: Ototoxicity M onitoring. *American Academy of Audiology* 2009; 1–25.

[14] Paken J, Govender CD, Pillay M, et al. Perspectives and practices of ototoxicity monitoring. *South African Journal of Communication Disorders* 2020; 67: 1–10.

[15] Garinis AC, Cornell A, Allada G, et al. Ototoxicity monitoring through the eyes of the treating physician: Perspectives from pulmonology and medical oncology. *International Journal of Audiology* 2018; 57: S19–S24.

[16] Singh M, Prasad CP, Singh TD, et al. Cancer research in India: Challenges & opportunities. *Indian J Med Res* 2018; 148: 362.

[17] D'cruz A, Yueh B, Das A, et al. Validation of the University of Washington quality of life questionnaires for head and neck cancer patients in India. Epub ahead of print 2007. DOI: 10.4103/0019-509X.39377.

[18] Tong A, Sainsbury P, Craig J. Consolidated criteria for reporting qualitative research (COREQ): A 32-item checklist for interviews and focus groups. *International Journal for Quality in Health Care* 2007; 19: 349–357.

[19] Rosenstock IM. Historical origins of the health belief model. *Health Educ Monogr* 1974; 2: 328–335.

[20] Sargeant J. Qualitative Research Part II: Participants, Analysis, and Quality Assurance. *J Grad Med Educ* 2012; 4: 1–3.

[21] Naderifar M, Goli H, Ghaljaie F. Snowball Sampling: A Purposeful Method of Sampling in Qualitative Research. *Strides in Development of Medical Education*; 14. Epub ahead of print 30 September 2017. DOI: 10.5812/sdme.67670.

[22] Saunders B, Sim J, Kingstone T, et al. Saturation in qualitative research: exploring its conceptualization and operationalization. *Qual Quant* 2018; 52: 1893–1907.

[23] Swain J. A Hybrid Approach to Thematic Analysis in Qualitative Research: Using a Practical Example. *A Hybrid Approach to Thematic Analysis in Qualitative Research: Using a Practical Example*. Epub ahead of print 2018. DOI: 10.4135/9781526435477.

[24] Chaibakhsh S, Zayeri F, Baghestani AR, et al. The effect of radiation therapy on hearing loss in patients with head and neck cancer. *Int J Cancer Manag*; 11. Epub ahead of print 2018. DOI: 10.5812/ijcm.10421.

[25] De Andrade V, Khoza-Shangase K, Hajat F. Perceptions of oncologists at two state hospitals in Gauteng regarding the ototoxic effects of cancer chemotherapy: A pilot study. *Afr J Pharm Pharmacol* 2009; 3: 307–318.

[26] Konrad-Martin D, Poling GL, Garinis AC, et al. Applying U.S. national guidelines for ototoxicity monitoring in adult patients: perspectives on patient populations, service gaps, barriers and solutions. *Int J Audiol* 2018; 57: S3–S18.

[27] Dille M. Barriers and Potential Solutions to Increase the Success of Ototoxicity Monitoring Programs. *ASHA*, https://www.asha.org/articles/barriers-and-potential-solutions-to-increase-the-success-of-ototoxicity-monitoring-programs/ (2018, accessed 8 November 2023).

[28] Peerbhay N, Munsamy DR, Dlamini HP, et al. The use of tele-audiology in ototoxicity monitoring: A scoping review. *J Telemed Telecare*. Epub ahead of print 2022. DOI: 10.1177/1357633X211068277.

Supporting Information

S1 Appendix 1. COnsolidated criteria for REporting Qualitative research (COREQ) Checklist

S2 Appendix 2. Interview guide for the semi-structured interviews

S3 Appendix 3. Participant quotes from the semi-structured interviews

Click here to access/download;Figure;Fig1.tif

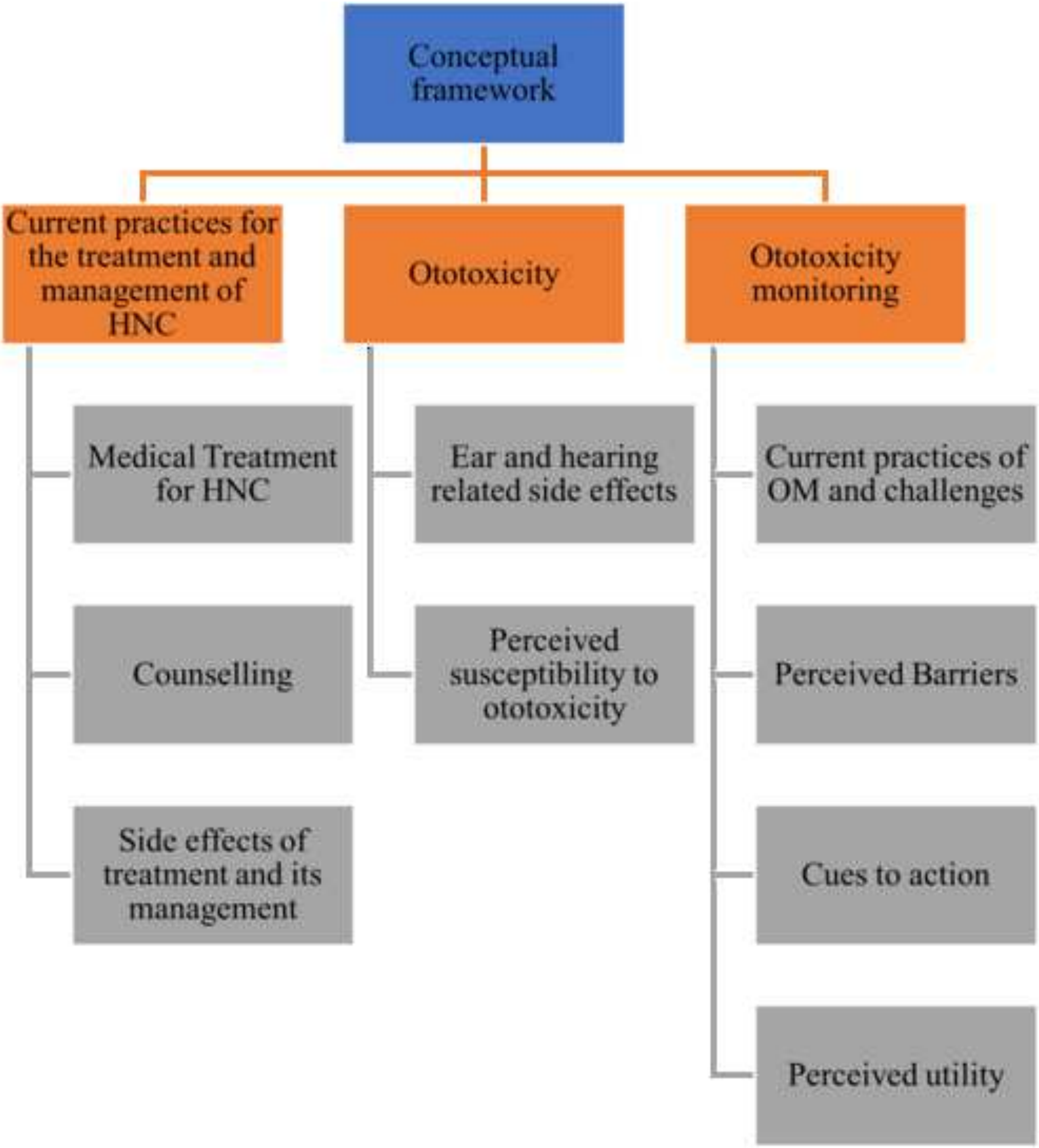

Click here to access/download
Supporting Information
S1_Appendix1.pdf

Click here to access/download
Supporting Information
S2_Appendix2.docx

Supporting Information S3 Appendix 3

Click here to access/download

Supporting Information

S3_Appendix3.docx
