# Supplementary material for: Oncologists’ views on ototoxicity monitoring in head and neck cancer patients: A South Indian qualitative study
Source: PLoS One. 2025 Jan 3;20(1):e0312847. doi: 10.1371/journal.pone.0312847 (PMC11698418; doi:10.1371/journal.pone.0312847)
Supplement: S2 Appendix — (DOCX) [file pone.0312847.s002.docx]

**Interview Guides**

**Interview Date: _________**

**Interview time: _________**

**Hello Dr…..**

I am Varsha Shankar, an Audiologist and Speech Language Pathologist working as a PhD Scholar at the Faculty of Audiology and Speech Language Pathology at SRIHER (DU).

I thank you for giving consent to participate in the study.

This interview is designed to understand the current practices, knowledge and perceptions of audiologists and oncologists about monitoring ototoxic effects in patients with head and neck cancer who are receiving CRT/ RT. The interview will last approximately 45 minutes to an hour. Before we begin, please let me know if you have any questions that I can answer or clarify.

The interview will now begin. I'm going to start audio recording our conversation for transcription and data analysis. The information will be kept strictly confidential, and your identity will never be revealed.

**Interview begins**

Dear Dr.___________

**Guides: (Probes have been italicized; constructs have been highlighted in yellow)**

1. How many years have you been practicing?
2. Describe your client base.
3. Please describe the current protocol followed at your hospital for the treatment of patients with head and neck cancer?	*(Current practices)* *(Chemotherapy – dosage, types of drugs, Radiation therapy – techniques used - IMRT, IGRT)*

***Only For Oncologists:***

4. How do you counsel patients regarding the treatment and its side effects?
5. *(Counselling regarding hearing loss, changes in balance and tinnitus especially before beginning the treatment)*	(*Current practices)*
6. Please describe some **common complaints** of patients with head and neck cancer undergoing RT/CRT? How do you deal with them?	*(Perceived susceptibility)*

(OR)

What are the possible side effects of these treatments?

When do you typically notice these symptoms and what type of medication were they on?

7. How important/serious are these complaints?	*(Perceived severity)*

*(How would the findings of these side effects* *influence treatment decisions?)*

How do you currently manage these complaints?

***Oncologists and Audiologists:***

8. What are ototoxicity monitoring programs (OMP)?
9. What is your opinion about OMP?	*(Benefits of OMP)*	*(Perceived benefits)*
10. Could you briefly describe the current status of OMP at your institution?

(*Current practices)*

*(On-going/ started and discontinued/ non-viable/ waiting for it to start as routine?)*

11. How does the referral process work in your institution?	(*Current practices)*

*(Do you refer all patients undergoing RT/CRT for audio-vestibular evaluation?)*

*(Do you refer patients selectively? If so, why? What in your opinion are the predictive factors used to refer patients undergoing RT/CRT for audio-vestibular evaluation?)*

*(Do you refer these patients for any other screening procedures?)*

12. Are you aware of any other such existing programs in Chennai? (*Current practices)*

*(If yes – probe for details, if no – probe for interviewee’s perception about* *why clinics have not implemented OMPs)*

13. What do you think are the key elements of an OMP?	*(Perceived barriers)*

(OR)

Could you describe any resources that can influence (either positively or negatively) the implementation of an OMP?

- *Financial/Economic*
- *Human - professional/ care takers/ technical help/ facilitator*
- *Infrastructure, Equipment/ technology, Technical/internet*
- *Organizational factors – acceptance, policy making/decision, SOP, Provider comfort/ ease/ acceptance/ perceptions?*
- *Patient’s comfort/ Acceptance/ Perceptions/ Sociocultural aspects/ Inherent patient related factors with respect to ear and hearing care?*
- *Motivators/ influencers*
- ***Evidence base for implementation of OMPs in the current context***
- *Any non-adaptable factors?*

14. Would you modify your current clinical practices to include OM? Why or why not?

*(Cues to action)*

15. If you plan on implementing an OMP, how confident are you regarding it’s sustainability? What are the challenges or facilitators you would expect?

*(Perceived barriers and Self-Efficacy)*

16. In your opinion, who are the role players in an OMP?

*How should the referral pathway be structured for an effective OMP?*

*Whose responsibility is it to provide patients with information about the possible ototoxic effects of medication?*

*What do you believe is the appropriate schedule of hearing monitoring tests for the results to be useful?*

Is there anything else you would like to say regarding ototoxicity and OM?

**Interview ends**

Thank you for your participation in the study. We will have the data analysed and will keep you updated regarding the progress of the study.
