# Supplementary material for: Oncologists’ views on ototoxicity monitoring in head and neck cancer patients: A South Indian qualitative study
Source: PLoS One. 2025 Jan 3;20(1):e0312847. doi: 10.1371/journal.pone.0312847 (PMC11698418; doi:10.1371/journal.pone.0312847)
Supplement: Attachment — Submitted filename: Response to Reviewers.docx [file pone.0312847.s004.docx]

6^th^ September, 2024

From,

Jayashree S

Associate Professor and Head

Department of Audiology

Sri Ramachandra Faculty of Audiology and Speech, Language Pathology

SRIHER (DU), Chennai, India

To,

The Editor

PLOS ONE

Dear Editor,

Sub: Response to reviewer’s comments on the manuscript - PONE-D-24-14073

I would like to thank you for considering our manuscript, “Current practices and perspectives of oncologists regarding ototoxicity monitoring in individuals with head and neck cancers undergoing radiation therapy and chemoradiotherapy: A qualitative study in a South Indian district” for publication in your esteemed journal. Upon the reviewer’s recommendation, we have changed the title to, “Oncologists' views on ototoxicity monitoring in head and neck cancer patients: A South Indian qualitative study.”

I take this opportunity to extend my gratitude to the reviewers for their valuable comments. Their suggestions and recommendations helped us improve our manuscript. We have answered all the reviewer’s comments in the document attached herewith. All recommendations have been incorporated in the manuscript and the changes are highlighted in yellow.

We look forward to hearing from you regarding the status of our submission.

Thanking you,

Sincerely,

Jayashree S

**Response to comments of Reviewer #1: Comments in the PDF file**

| **Reviewer query/comment** | **Response to the reviewer** | **Page no. and line no.** |
| --- | --- | --- |
| Line 50 Surgical oncologist? Medical Oncologist? - Write in full | This change has been incorporated as recommended by the reviewer. | Page 3, Line 50 |
| Line 58 Write in full at the beginning of the sentence | This sentence has been rephrased for better grammar and readability. | Page 3, Line 58 |
| Line 100-101 What research approach did the researcher employ? | The study used a qualitative approach and a cross-sectional snapshot study design was used. This has been added in the method. | Page 6, Lines 100 – 104 |
| Line 99-100 include a section maybe after data analysis to discuss the ethical considerations or issues. | This change has been incorporated as recommended by the reviewer. A section on ethical considerations has been elaborated after the data analysis in the method section | Page 9, Lines 177 - 184 |
| Line 111 Where? not the actual centers but the area (Country/Province/State). | We thank the reviewer for this valuable comment. This change has been incorporated as recommended by the reviewer. | Page 7, Line 125 |
| Line 112 How? This makes it seem as if the researcher provided some form of intervention to 'ensure' the participants were knowledgeable. I suggest working on the sentence construction here for clarity if this was part of the inclusion criteria | We thank the reviewer for this valuable comment. This sentence has been edited accordingly as recommended by the reviewer. | Page 7, Lines 126 - 128 |
| Line 117 How were the participants contact details obtained? | The researchers obtained the contact numbers of several oncologists through known contacts and colleagues in the field of medical and radiation oncology. This information has been added as recommended by the reviewer. | Page 7, Lines 132 - 133 |
| Under interview details, were there no field notes take during the on-site interviews? | The interviews were audio recorded and field notes were maintained during the interviews. This has been mentioned in the method. | Page 8, Lines 148 - 149 |
| Line 135 What does QDA stand for? write in full, since this is the only place intext where the acronym is used | QDA stands for Qualitative Data Analysis. This change has been incorporated as recommended by the reviewer. | Page 8, Lines 155- 156 |
| Line 154 I suggest tabulating the information that follows. Also tabulate this data to clearly indicate if the bulleted points represent sub-themes or categories. | We thank the reviewer for this valuable comment. This change has been incorporated as recommended by the reviewer. | Page 12, Line 205 |
| Results: I suggest including the quotes in text. The current format is not reader friendly and is very confusing. Suggested read: Cristancho, S., Watling, C.J. and Lingard, L.A. 2021. Three principles for writing an effective qualitative results section. Focus on Health Professionals, VOL. 22, NO. 3, 111- 124 | We thank the reviewer for this valuable comment. The quotes have been included in the results section. | Pages 12 - 38 |
| Line 175 I suggest including quotes in the text | This change has been incorporated as recommended by the reviewer. | Pages 12 - 38 |
| Line 191 I suggest including quotes in the text | This change has been incorporated as recommended by the reviewer. | Pages 12 - 38 |
| Line 459 which countries are being referenced? | The authors referenced to the United States of America in this statement. The statement has been edited accordingly. | Page 42, Line 979 |
| Line 145 discuss limitations separately and elaborate how the limitations impacted the study. | We thank the reviewer for this valuable comment. This change has been incorporated as recommended by the reviewer. | Page 44, Lines 1023 - 1029 |

**Response to comments of Reviewer #1:**

| **Reviewer query/comment** | **Response to the reviewer** | **Page no. and line no.** |
| --- | --- | --- |
| The sectional headings should be arranged correctly. | This change has been incorporated as recommended by the reviewer. The sectional headings are arranged according to the COREQ checklist. | Pages 6 - 10 |
| There critical information missing from the manuscript. The authors should describe the setting in enough detail to give the reader a clear picture. | This change has been incorporated in the methods section as recommended by the reviewer. In addition, table 1 provides details regarding the setting of the interview participants who consented to participate in the study. | Page 8, Lines 141 - 143 |
| The research approach should be highlighted in order to allow the reader to identify the coherence in the methods used in the study. | The study incorporated a qualitative research approach. This has been mentioned in the methods section. | Page 6, Lines 100 - 104 |
| The authors should include a section to clearly describe all ethical considerations / issues pertaining to the study. | This change has been incorporated in the methods section after data analysis as recommended by the reviewer. | Page 9, Lines 177 - 184 |
| Furthermore, the authors should clarify the inclusion and exclusion criteria instead of stating that they ensured certain qualities existed within the selected population. | This change has been incorporated as recommended by the reviewer. | Page 7, Lines 126 - 131 |
| The results section should be reorganized as the current form does not read well. All excerpts must be included in text to show how the quotations fit in the narrative element. I have shared an article in the track changes to guide and support this. | This change has been incorporated as recommended by the reviewer. | Pages 12 - 38 |
| The discussion could benefit form more literature support as there is large pieces of paragraphs where the authors have just written form the heart. | This change has been incorporated as recommended by the reviewer. The discussion has been edited to include more information and literature support. | Pages 38 - 44 |
| The authors should create a separate heading for the limitations and elaborate on how these limitations impacted their study. | This change has been incorporated as recommended by the reviewer. | Page 44, Lines 1023 - 1029 |
| Include a conclusion to provide a summary of the whole study. | This change has been incorporated as recommended by the reviewer. | Page 44, Lines 1030 - 1039 |

**Response to comments of Reviewer #2:**

| **Reviewer query/comment** | **Response to the reviewer** | **Page no. and line no.** |
| --- | --- | --- |
| Title: Consider shortening the title of your study to less than 15 words. A shorter title can better grab readers' attention and convey the main idea concisely. My suggestions could be;  1. “Oncologists' views on ototoxicity monitoring in head and neck cancer patients: A South Indian qualitative study”  2. “Exploring Ototoxicity Awareness Among Oncologists in South Indian Cancer Care” | This change has been incorporated as recommended by the reviewer. The current title is now, “Oncologists' views on ototoxicity monitoring in head and neck cancer patients: A South Indian qualitative study”. | Page 1, Line 1 |
| Abstract: Methods – COREQ is an abbreviation, in this instance, it has been used without a prior definition. State in full before using it. | This change has been incorporated as recommended by the reviewer. | Page 2, Line 23 |
| Keywords: Is “semi-structured” appropriate as a keyword? | “Semi-structured” has been removed from the keyword list. | Editorial Manager Portal; Manuscript data; Keywords |
| Line 41 – grammatical error “In India, head and neck cancers (HNC) are one” not “is” | This change has been incorporated as recommended by the reviewer. | Page 3, Line 41 |
| Lines 45-47 – the sentence is too long and loses its meaning. Consider breaking it down into two or rephrasing it | This change has been incorporated as recommended by the reviewer. | Page 3, Lines 45 - 47 |
| Line 51 – grammatical error “the” missing, treatment depends upon the stage | This change has been incorporated as recommended by the reviewer. | Page 3, Line 51 |
| Line 58 – Avoid starting a sentence with an abbreviation | This change has been incorporated as recommended by the reviewer. | Page 3, Line 58 |
| The COREQ guidelines mandate including a section that identifies the research design and provides the rationale for its selection. | This change has been incorporated as recommended by the reviewer. | Page 6, Lines 100 - 104 |
| Line 111 – The abbreviation for ROs has already been defined, no need to repeat it here | This change has been incorporated as recommended by the reviewer. | Page 7, Line 125 |
| Interview details – Could you please provide a list of the interview questions that were included in the interview guide? | The interview guide has been provided as figures 2 and 3, in addition to the appendix file (S2 Appendix 2). | Page 8, Lines 145, 152, Figs 2 and 3, S2 Appendix 2 |
| Analysis – To provide clarity, it would be beneficial to specify the approach used in thematic analysis, such as employing the method outlined by Braun and Clarke. Thematic analysis is too broad | This change has been incorporated as recommended by the reviewer. | Page 8, Lines 155 - 172 |
| Providing a citation and a brief explanation of what QDA Miner Lite is would be beneficial for the readers. | This change has been incorporated as recommended by the reviewer. | Page 8, Lines 155 - 157 |
| While efforts have been made to describe measures for ensuring the trustworthiness of the study, it is recommended to have a dedicated section that explicitly outlines this process. Providing a detailed explanation of how your study, for instance, addresses all four criteria for assessing trustworthiness, as outlined by Lincoln and Guba, will enhance the credibility of the methods section. | We thank the reviewer for this interesting suggestion. This change has been incorporated as recommended by the reviewer. The measures to ensure trustworthiness has been described in the methods section. | Page 10, Lines 185 - 198 |
| I am curious about how to navigate this section. Will readers need to refer back to the quotes while simultaneously reading the results section? Why not follow the conventional approach of integrating the quotes within the results section for readability? | This change has been incorporated as recommended by the reviewer. The quotes have been integrated within the results section for readability. | Pages 12 - 38 |
| Lines 221 and 227 – Avoid starting sentences with abbreviations. Correct this throughout the document | This change has been incorporated as recommended by the reviewer. | Page 17, Line 340 |
| Lines 402-420, feel like a rehash of the findings as there is no attempt to link the stated findings to existing literature. | This change has been incorporated as recommended by the reviewer. The discussion has been edited to include more information and literature support. | Pages 39 - 40 |
| Line 421 – “Contrary to beliefs of oncologists” Which oncologists? From this study or? The statement further says numerous studies but only one study is cited. Please cite those studies | This sentence has been edited for better flow and readability.  The subsequent statement regarding numerous studies has been edited to include the citations of the respective studies, | Page 39, Lines 912 - 916 |
| Line 422 – include the names of the authors of the systematic review. Like “A systematic review by Theunissen et al., …” | This change has been incorporated as recommended by the reviewer. | Page 40, Line 916 |
| Line 426 – “This finding aligns with previous studies [14]” but only one study is cited, please cite the studies otherwise change to “This finding aligns with a previous study [14]” | This change has been incorporated as recommended by the reviewer. | Page 41, Line 941 |
| There is a need for a discussion on how the study’s findings align or misalign with the theoretical framework. Furthermore, suggestions for further research on how the framework employed can be improved are needed. | This change has been incorporated in the discussion as recommended by the reviewer. | Page 43, Lines 1005 - 1013 |
| There is no attempt to explore potential avenues for future research in general | This change has been incorporated as recommended by the reviewer. The future directions of the study have been elaborated in the discussion. | Page 43, Lines 1014 - 1019 |
| Line 475-481 – there is no attempt to link the findings to existing literature | This change has been incorporated as recommended by the reviewer. The discussion has been edited to include more information and literature support. | Pages 43, Lines 995 – 1004 |
