# Supplementary material for: Oncologists’ views on ototoxicity monitoring in head and neck cancer patients: A South Indian qualitative study
Source: PLoS One. 2025 Jan 3;20(1):e0312847. doi: 10.1371/journal.pone.0312847 (PMC11698418; doi:10.1371/journal.pone.0312847)
Supplement: Attachment — Submitted filename: Response to Reviewers.docx [file pone.0312847.s006.docx]

9^th^ October, 2024

From,

Jayashree S

Associate Professor and Head

Department of Audiology

Sri Ramachandra Faculty of Audiology and Speech, Language Pathology

SRIHER (DU), Chennai, India

To,

The Editor

PLOS ONE

Dear Editor,

Sub: Response to reviewer’s comments on the manuscript - PONE-D-24-14073

I would like to thank you for considering our manuscript, “Oncologists' views on ototoxicity monitoring in head and neck cancer patients: A South Indian qualitative study” for publication in your esteemed journal.

I take this opportunity to extend my gratitude to the reviewers for their valuable comments. Their suggestions and recommendations helped us improve our manuscript. We have answered all the reviewer’s comments in the document attached herewith. All recommendations have been incorporated in the manuscript and the changes are highlighted in yellow.

We look forward to hearing from you regarding the status of our submission.

Thanking you,

Sincerely,

Jayashree S

**Response to comments of Reviewer #1: Comments in the PDF file**

| **Reviewer query/comment** | **Response to the reviewer** | **Page no. and line no.** |
| --- | --- | --- |
| Line 23 include the correct research design. COREQ is not a research design, it is a checklist for reporting of studies using interviews and focus groups. | This change has been incorporated as recommended by the reviewer. | Page 2, Lines 25 - 26 |
| Line 103 Use this as the first sentence | This change has been incorporated as recommended by the reviewer. | Page 7, Lines 108 - 109 |
| Line 157 Remove (2006, 2012) | This change has been incorporated as recommended by the reviewer. | Page 9, Line 163 |

**Response to comments of Editor:**

| **Reviewer query/comment** | **Response to the reviewer** | **Page no. and line no.** |
| --- | --- | --- |
| I recommend starting the abstract with a background or introduction and ending it with a conclusion instead of a discussion section. | This change has been incorporated as recommended by the editor. The sectional headings are arranged according to the COREQ checklist. | Pages 2 - 3 |
| The materials and methods section is started with ethical permission. This statement may be placed under the ethical consideration heading. There is no connectivity between the sentences in the first paragraph. Organize the materials and methods section to maintain connectivity between the sentences and paragraphs. | This change has been incorporated in the methods section as recommended by the reviewer.  The method of the study is presented according to sectional headings of the COREQ checklist such as, “Research team and reflexivity”, “Study design”, “Analysis and findings”. | Pages 7 - 11 |
